# Expression of an endoglucanase–cellobiohydrolase fusion protein in *Saccharomyces cerevisiae, Yarrowia lipolytica,* and *Lipomyces starkeyi*

**DOI:** 10.1186/s13068-018-1301-y

**Published:** 2018-12-03

**Authors:** Qi Xu, Markus Alahuhta, Hui Wei, Eric P. Knoshaug, Wei Wang, John O. Baker, Todd Vander Wall, Michael E. Himmel, Min Zhang

**Affiliations:** Biosciences Center, National Renewable Energy Laboratory, Golden, CO 80401 USA

**Keywords:** Fusion protein, Oleaginous yeast, CBHI, Consolidated bioprocessing, Cellulase, Cellobiohydrolase

## Abstract

The low secretion levels of cellobiohydrolase I (CBHI) in yeasts are one of the key barriers preventing yeast from directly degrading and utilizing lignocellulose. To overcome this obstacle, we have explored the approach of genetically linking an easily secreted protein to CBHI, with CBHI being the last to be folded. The *Trichoderma reesei* eg2 (*Tr*EGII) gene was selected as the leading gene due to its previously demonstrated outstanding secretion in yeast. To comprehensively characterize the effects of this fusion protein, we tested this hypothesis in three industrially relevant yeasts: *Saccharomyces cerevisiae, Yarrowia lipolytica,* and *Lipomyces starkeyi*. Our initial assays with the *L. starkeyi* secretome expressing differing *Tr*EGII domains fused to a chimeric *Talaromyces emersonii*–*T. reesei* CBHI (*TeTr*CBHI) showed that the complete *Tr*EGII enzyme, including the glycoside hydrolase (GH) 5 domain is required for increased expression level of the fusion protein when linked to CBHI. We found that this new construct (*Tr*EGII–*TeTr*CBHI, Fusion 3) had an increased secretion level of at least threefold in *L. starkeyi* compared to the expression level of the chimeric *TeTr*CBHI. However, the same improvements were not observed when Fusion 3 construct was expressed in *S. cerevisiae* and *Y. lipolytica*. Digestion of pretreated corn stover with the secretomes of *Y. lipolytica* and *L. starkeyi* showed that conversion was much better using *Y. lipolytica* secretomes (50% versus 29%, respectively). In *Y. lipolytica*, *TeTr*CBHI performed better than the fusion construct. Furthermore, *S. cerevisiae* expression of Fusion 3 construct was poor and only minimal activity was observed when acting on the substrate, *p*NP-cellobiose. No activity was observed for the *p*NP-lactose substrate. Clearly, this approach is not universally applicable to all yeasts, but works in specific cases. With purified protein and soluble substrates, the exoglucanase activity of the GH7 domain embedded in the Fusion 3 construct in *L. starkeyi* was significantly higher than that of the GH7 domain in *TeTr*CBHI expressed alone. It is probable that a higher fraction of fusion construct CBHI is in an active form in Fusion 3 compared to just *TeTr*CBHI. We conclude that the strategy of leading *TeTr*CBHI expression with a linked *Tr*EGII module significantly improved the expression of active CBHI in *L. starkeyi*.

**Electronic supplementary material:**

The online version of this article (10.1186/s13068-018-1301-y) contains supplementary material, which is available to authorized users.

## Background

Cellulose and lignin present in biomass are the most abundant forms of organic carbon on Earth. As such, biofuels produced from lignocellulose-derived sugars represent one of the most promising alternative energy sources to date. The primary bottleneck in the production of lignocellulosic biofuels is the high cost associated with release of monomeric sugars for fermentation due to recalcitrance of the plant cell wall. To overcome this difficulty, consolidated bioprocessing (CBP) has been proposed in which cellulose degradation and biofuel production are combined in a single microorganism [1, 2]. Microbial CBP cells perform these processing steps in a single fermenter with the benefit of reducing the feedback inhibition of released monomeric sugars to cellulases, therefore reducing the cost of biofuel production.

Among microorganisms, yeasts are promising potential CBP candidates, as they are well known to rapidly produce high yields of biofuels or their precursors, e.g., ethanol in *Saccharomyces cerevisiae*, and fatty acids or lipids from *Yarrowia lipolytica* [3, 4], *Lipomyces starkeyi* [5–7], and *S. cerevisiae* [8]. Yeasts, however, cannot degrade cellulose directly due to their general lack of aggressive cellulase systems [1, 9]. To solve this problem, significant expression and secretion levels of heterologous cellulases in yeasts must be achieved. Of particular importance is cellobiohydrolase I (CBHI), an essential cellulase in fungal cellulase systems [9, 10]. Previous research in developing yeast CBP organisms has focused on *S. cerevisiae* [1, 11, 12], *Y. lipolytica,* [13–15], and *L. starkeyi* [16]. However, these previous studies of the expression of fungal CBHIs in yeast have encountered major challenges, such as low secretion, yield, and activity of the recombinant proteins which directly discourages the application of these yeast to CBP [17, 18]. Clearly, low secretion levels of heterologous CBHI are one of the key obstacles for yeast CBP that must be resolved. Some fungal cellulases, such as *T. reesei* endoglucanase II (*Tr*EGII), have been reported to have high secretion levels (compared to CBHI enzymes) in *L. starkeyi* [16]. Specifically, we reported previously that the secretion level of *Tr*EGII in *L. starkeyi* is much higher than that of a chimeric CBHI generated by the fusion of the catalytic module from *Talaromyces emersonii* CBHI with the linker peptide and cellulose-binding module from *T. reesei* CBHI (*TeTr*CBHI) [16].

Cellulase enzymes are commonly multi-modular (CaZy database, [19]), indicating that nature is already using the concept of fused enzymes to improve properties. Examples range from those that remain somewhat ambiguous, such as X modules (also called “FnIII-like” domains [20–22]), to carbohydrate binding modules (CBMs) that guide substrate binding [23] to enzymes containing multiple catalytic modules [24–26]. Beyond natural occurrences, some positive results have been demonstrated by linking a leading CBM module to CBHI [27]. Dockerin containing enzymes have also been incorporated into artificial cellulosomes resulting in enhanced synergetic activity [28]. Other than carbohydrate active enzymes, fusions of high yield proteins, such as ubiquitin, have been used to increase expression levels [29].

Thus, to overcome the low secretion levels of heterologous CBHI in yeasts, we have linked the well secreted *Tr*EGII or its individual modules (CBM1 and GH5), as a leading protein to enhance the secretion of chimeric *TeTr*CBHI. In this paper, we present the results of the secretion and activities of various fusion enzymes compared to the individual *Tr*EGII and *TeTr*CBHI enzymes in three potential CBP yeast candidates, *S. cerevisiae*, *Y. lipolytica,* and *L. starkeyi*.

## Methods

Yeast strains were grown in YPD at 30 °C (with shaking at 220 rpm) for general growth and transformation unless noted. The Gibson Assembly Cloning Kit (NEB, Ipswich, MA, USA) was used to insert target genes into vectors [16] and then followed by standard gene cloning protocols [30]. The primers and plasmids used in this study are listed in Additional files 1 and 2, respectively.

*Lipomyces starkeyi* NRRL Y-11557 was acquired from the ARS Culture Collection (NRRL) and was transformed following the optimized transformation protocol as described [31] and modified by Xu et al. [16]. Briefly, cells were grown to an OD_600_ of approximately 10. The transformation mixture consisted of 240 μL 50% PEG 3650, 30 μL 1.0 M lithium acetate, 15 μL ssDNA, and 15 μL of DNA in water and a final volume of 350 μL (including cells). Cells were heat shocked in a 40 °C water bath for 5 min and incubated at 30 °C for 3 h before being plated on YPD plates with 30 μg/mL of clonNAT. For *Y. lipolytica*, the gene coding sequences for single cellulases (i.e., *Tr*EGII and *TeTr*CBHI) were codon-optimized based on the codon bias of *Y. lipolytica* and were synthesized by GenScript (Piscataway, New Jersey, USA) and described in detail previously [15].

*Yarrowia lipolytica* strain Po1g (MatA, leu2-270, ura3-302:URA3, xpr2-332, axp-2) and secretion vector pYLSC1 were acquired from Yeastern Biotech Co. (Taipei, Taiwan). The *Y. lipolytica* secretion vector (pYLSC1) contained a hybrid promoter (hp4d), a secretion signal of alkaline extracellular protease (XPR2), XPR2 terminator, and the LEU2 selection marker. The Fusion 3 construct was amplified using the primers 158-F and 158-R. *Y. lipolytica* was transformed with NotI-linearized Fusion 3 construct using *YLOS* One Step Transformation system and the *YLEX* expression kit (Yeastern Biotech Co., Taipei, Taiwan), as described previously [15]. Note that NotI digestion linearized the construct’s plasmid DNA (in the pBR region) to create a linear DNA fragment that is capable of inserting into the pBR docking platform of the recipient Po1g strain; thus, it is targeted integrative transformation. The transformation mixture was spread on YNB selection plates for Leu^+^ colonies of transformants.

The replicating Fusion 3 expression plasmid for *S. cerevisiae* was built on pD1214 which has the strong constitutive TEF promoter, 11 different secretion signal sequences, CYC terminator, and URA3 marker for selection (https://www.atum.bio/products/expression-vectors/yeast). The gene encoding the Fusion 3 protein was amplified via PCR using primers EK243 and EK246 having the electra overlap ends (Additional file 1) for rapid cloning into the pD1214 vector. This generated 12 different plasmids (Additional file 2) which were transformed into BFY716 (*S. cerevisiae* D5A *ura3*::APH 3′ II, *ura3*::HPH) for expression. *S. cerevisiae* was transformed as previously described [8]. Transformants were grown on YNB media without uracil and containing glucose as the carbon source.

### Genomic DNA extraction and estimation of transgene copy number

Genomic DNA of the selected transformants was isolated from *L. starkeyi* cell pellets using the procedure described previously [16]. Transgene copy number was estimated using a real-time qPCR method described by Weng et al. [32], which had been used in numerous studies [33–35] and is described in our recent publication [16]. Briefly, the target gene (X) copy number was calculated versus the reference gene (R) by X_o_/R_o_. The endogenous eukaryotic initiation factor 5 (eif5) gene was used as a single copy reference gene [36]. The primers for the reference gene and the target gene are listed in Additional file 1.

### Comparison of protein secretion abundance using a Western blot

Transformant colonies were re-streaked for isolation. Single colonies were picked from the re-streaks of the transformants and grown in YPD until the OD_600_ reached 15. Cultures were standardized to the same optical density and the cells removed by centrifugation at 5000×*g*. The resulting supernatants were used directly for Western blot analysis. The densitometry analysis for the relative intensity of Western blot bands was conducted using Quantity One analysis software (Bio-Rad Laboratories, Hercules, CA).

### Endo-cellulase activity with Congo red staining

*Lipomyces starkeyi* culture supernatant (20 μL) was spotted on a plate containing 1% agarose (Sigma-Aldrich Corp., St. Louis, MO, USA) supplemented with 0.5% carboxymethylcellulose (CMC, Sigma-Aldrich Corp., St. Louis, MO, USA) and incubated at 37 °C overnight. Eight milliliter of 0.1% Congo red was added to the plate and shaken gently for 30 min on a rocker. The Congo red stain was decanted, and the stained plate was de-stained with 1 M NaCl and then shaken for 30 min. The de-staining step was repeated two more times with water. Water titrated to pH 2.0 with HCl was added to darken the Congo red dye immediately before taking photographs.

### Fermentation

Production of all protein constructs was carried out in a 14-L BioFlo 310 bioreactor (New Brunswick Scientific—Eppendorf, Edison, NJ) in a 10-L culture. All seed cultures were inoculated from a single colony into 50 mL of YPD medium in a 250-mL flask, incubated at 30 °C and 225 RPM, and then transferred after 24 h of incubation to 1 L of fresh YPD medium (pH 5.0) in a 2.8-L baffled flask. The secondary seed culture was subsequently transferred into the fermenter after approximately 36 h of incubation. The *L. starkeyi* fermentations were controlled at 30 °C, 300 RPM, one volume of air per volume of media per min (VVM) at pH 5.2 in YPD medium containing 5% glucose. The fermentation was run until OD_600_ reached maximum, usually between 72 and 96 h. *Y. lipolytica* fermentations were controlled at 28 °C, 300 RPM, one VVM air with extra baffling at pH 5.0 in YPD containing 50 mM citrate buffer (2:1 citric acid/sodium citrate) for increased buffering. The fermentation was run until a maximum OD_600_ value was reached, usually between 72 and 120 h. *S. cerevisiae* fermentations were controlled at 30 °C, shaking at 300 RPM, and one VVM air in YNB-ura pH 5.0 medium containing 5% glucose. The fermentation was run for approximately 24 h, at which point all glucose was consumed. All culture broths were pelletized via centrifugation and concentrated using tangential flow ultrafiltration with a 10,000 kDa MWCO membrane. The concentrated culture broths were buffer exchanged into 20 mM Bis–Tris pH 6.5 in preparation for column chromatography.

### Protein purification

After concentration and buffer exchange, the proteins were further purified. First, the ammonium sulfate concentration of the sample was slowly adjusted to 1.5 M at 4 °C and filtered with a 0.45-μm Nalgene Rapid-Flow Bottle Top filter (Thermo Scientific Pierce Protein Biology Products, Rockford, IL, USA). Then, the eluate was applied to a GE XK 26 column packed with hydrophobic interaction chromatography resin (Phenyl Sepharose 6 fast flow) and equilibrated with a buffer containing 50 mM Bis–Tris pH 6.5 and 1.5 M ammonium sulfate. The partially purified protein was then eluted out with a descending ammonium sulfate gradient and desalted in 20 mM Bis–Tris pH 6.5 buffer using two HiPrep 26/10 Desalting columns in series. Next anion exchange chromatography with a Tricorn 10/100 anion exchange column packed with Source 15Q resin in 20 mM Bis–Tris pH 6.5 and an increasing NaCl gradient was used before final purification with size exclusion chromatography using a GE 26/60 Superdex 75 column in 20 mM acetate pH 5.0 and 100 mM NaCl buffer. Whenever necessary, Vivaspin 20 10 kDa concentrators were used to concentrate the samples, and the desired protein fractions were identified using *p*-nitrophenyl-β-lactoside (*p*NP-lactose) assay [37]. All chromatography columns, resins, and concentrators were purchased from GE Healthcare (Piscataway, NJ, USA). Protein purity was assessed by SDS-PAGE, and concentration was determined using A_280_.

### Enzyme activity assays

Cellulase activity was measured using dilute acid-pretreated corn stover (PCS) or Avicel (Avicel PH-101, Fluka; Sigma-Aldrich Corp., St. Louis, MO) as a substrate. The PCS used was NREL dilute acid-pretreated corn stover P050921, produced in a vertical pulp digester supplied by Sunds Defibrator (now Metso Corporation, Helsinki, Finland) as described earlier [38], with a residence time of approximately one min at 190 °C and 0.45 g H_2_SO_4_ per g dry biomass at 30% solids loading, yielding pretreated solids containing 59.1% in glucan, 5.1% in xylan, and 25.3% in lignin. Because the theoretical molecular weight of the *TeTr*CBHI is slightly greater than that of the *Tr*CBHI, an equal molar loading resulted in a loading of 25.0 mg/g cellulose for the *TeTr*CBHI, compared to 24.6 mg/g cellulose for the *Tr*CBHI. For the PCS substrate, a loading of 5.7 mg/mL was used. In addition to CBHI, some assays also contained the catalytic domain of E1 (with Y245G mutation) from *Acidothermus cellulolyticus* or *Tr*EGII at 1.7 mg/g cellulose. All assays included *A. niger* beta glucosidase (BGL), which was chromatographically purified from the commercial mixture Novozyme 188 (Novozymes North America, Franklinton, NC, USA). BGL was loaded into the reaction mixtures at a concentration of 0.4 mg/g of cellulose substrate.

Assays were carried out at 40 °C in 20 mM acetate, pH 5.0 containing 0.02% (w/v) sodium azide to inhibit microbial growth. Assays were done in triplicate, in initial volumes of 1.7 mL in crimp-sealed 2.0-mL HPLC vials, with constant mixing by inversion at 10 times per min in a 40 °C water bath. At designated time points during the digestions, representative 0.1-mL aliquots of liquid and solids were withdrawn for analysis. The withdrawn aliquots of the digestion mixture were diluted 18-fold with deionized water into sealed 2.0-mL HPLC vials and then immersed for 10 min in a boiling water bath to terminate the enzyme reactions. The diluted aliquots were filtered with 0.2-μm filter before the determination of released sugars by HPLC, as described previously [15].

### *p*NP-lactose and *p*NP-cellobiose assays

Activity with *p*NP-lactose or *p*NP-cellobiose as a substrate was measured using a Molecular Devices Spectra MAX 190 spectrophotometer. For each assay, 80 μL of 2 mM substrate in 50 mM acetate pH 5.0 was added to each well of a 96-well plate, followed by 20 μL of each protein fraction or culture supernatant. The plate was then incubated 30 min at 45 °C. The reactions were quenched with 20 μL 1.0 M NaOH, and the absorbance at 405 nm was measured.

## Results and discussion

### Fusion construct evaluation in *L. starkeyi*

**Fig. 1:**
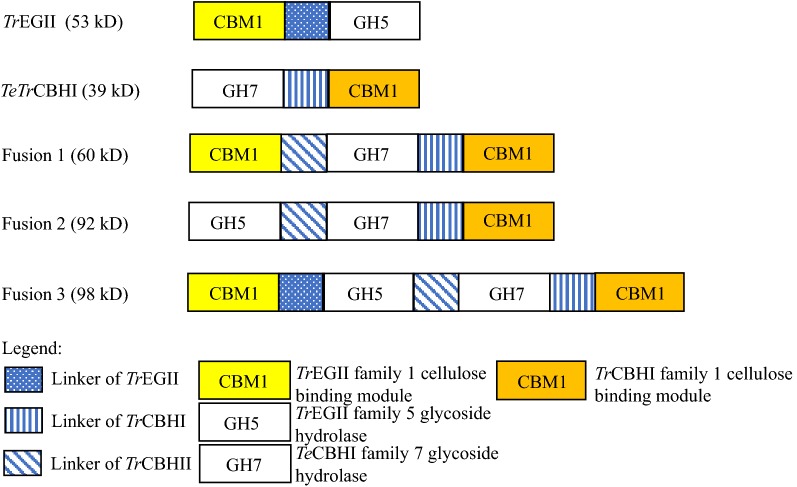
Protein domain structure of the native enzymes and fusion constructs in the N-terminal to C-terminal orientation. Fusion 1, 2, and 3 constructs have *Tr*EGII or its individual modules connected to the N-terminal end of *TeTr*CBHI using a *Tr*CBHI linker. Yellow indicates the CBM1 of *Tr*EGII, orange indicates CBM1 of *TeTr*CBHI, blue indicates linkers, and white indicates catalytic modules

To sustain growth on cellulosic biomass, the minimal cellulase activities required include a β-d-glucosidase, an endoglucanase, and a cellobiohydrolase [9]. Cellobiohydrolases are a major component of the *T. reesei* secretome when growing on biomass [39]. However, expressing sufficient levels of active enzyme in yeasts is challenging [1, 9]. To overcome this limitation, we designed a series of fusion constructs linking the various modules of EGII to *TeTr*CBHI (Fig. 1). Previously, we observed high levels of secretion of *Tr*EGII compared to *TeTr*CBHI in *L. starkeyi* [16]. The higher secretion of *Tr*EGII could be due to it being relatively easier to fold in yeasts than the native CBHI. Other explanations, such as hyper-glycosylation, protease degradation, and incompatibility with the host genetic system are also possible [27, 40]. However, it is also known that yeasts specifically have problems with correctly forming CBHI disulfide bonds [17]. Based on this finding, we hypothesized that *Tr*EGII could be used to enhance CBHI secretion in yeast by leading translation with an easy-to-fold protein that could aid in the correct folding of the target protein and thus successfully secrete a more complex protein. A similar experiment using a leading CBM module has produced somewhat positive results before [27]. To test our hypothesis, we proposed that the full-length *Tr*EGII or its individual domains could be fused with CBHI at the N-terminus to generate fusion proteins. Three fusion genes were designed and constructed (Fig. 1). Fusion 1 was generated by fusing the CBM1 domain of *Tr*EGII with the full-length *TeTr*CBHI. Fusion 2 was generated by fusing the GH5 domain of *Tr*EGII to the full-length *TeTr*CBHI and Fusion 3 contained the full-length *Tr*EGII fused with the full-length *TeTr*CBHI. In all the fusion proteins tested, the additional *Tr*EGII domains were tethered to *TeTr*CBHI by the 41 amino acid *T. reesei* CBHII linker peptide.

### Fusion construct secretion in *L. starkeyi*

**Fig. 2:**
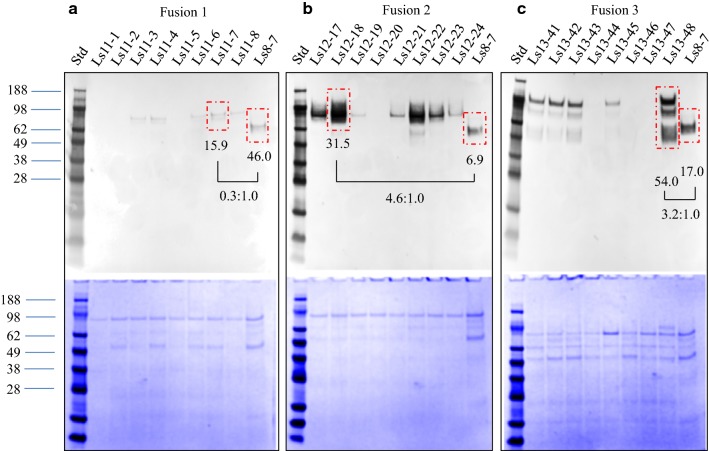
Screening of eight secretomes of *L. starkeyi* transformants for each fusion protein by Western blot. The top panel shows a Western blot, and the bottom panel shows the corresponding SDS-PAGE gel. **a** The secretomes from transformants expressing Fusion 1, **b** for Fusion 2, and panel C for Fusion 3. The lane having Ls8-7 shows the secretome from a transformant having the individual *TeTr*CBHI construct. Further notes regarding the methodology and validity of conclusions based on this figure can be found in Additional file 3

**Fig. 3:**
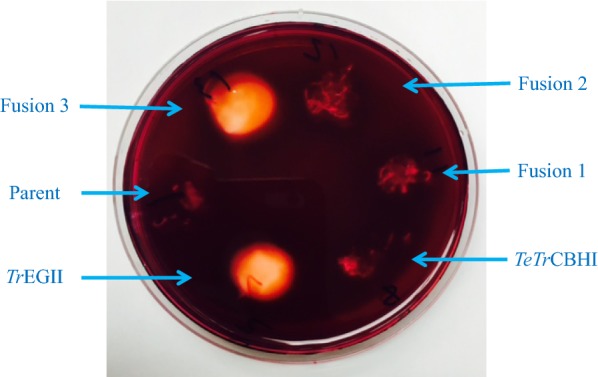
*L. starkeyi* secretome endoglucanase activity with Congo red staining. Secretomes, as culture supernatant from transformant cultures, were used directly for Congo red testing. *Tr*EGII, *TeTr*CBHI, Fusion 1, Fusion 2, and Fusion 3 show secretomes from transformants integrated with *Tr*EGII, *TeTr*CBHI, Fusion 1, Fusion 2, or Fusion 3 constructs, respectively. Parent indicates the WT *L. starkeyi* transformant with empty vector

**Fig. 4:**
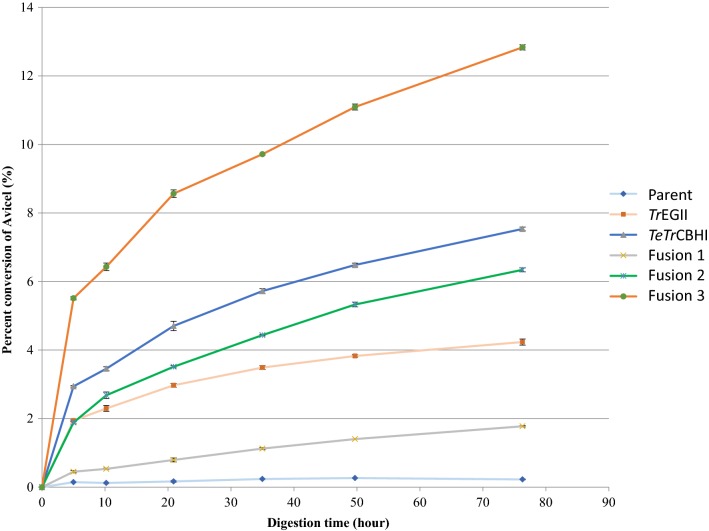
*L. starkeyi* secretome activity on Avicel. All secretome proteins were loaded at 28 mg of overall secretome protein, along with 0.5 mg of purified *A. niger* β-glucosidase per g cellulose. The enzymes could act on 5 mg cellulose per mL, at 40 °C, pH 5.0 in 20 mM acetate. Conversion percentages were calculated based on HPLC determinations of released monomeric glucose

The expression vectors listed in Additional file 2 were transformed into *L. starkeyi,* and positive transformants (clonNAT resistant colonies) were grown on YPD. The expression and secretion of these transformants was tested at the secretome level for yield (Fig. 2 and Additional file 3), endoglucanase activity—using CMC with Congo red staining (Fig. 3), and Avicel digestion assays (Fig. 4). Cell-free culture supernatants were used directly for SDS-PAGE and Western blot analysis using monoclonal antibody directed toward the linker-CBM1 region of *Tr*CBHI (Fig. 2a–c). Figure 2a shows the expression of the Fusion 1 (CBM1 domain of *Tr*EGII fused with *TeTr*CBHI) with eight single positive colonies resistant to clonNAT (Ls11-1 to Ls11-8). Five colonies showed positive bands on a Western blot, indicating Fusion 1 could be expressed and secreted in *L. starkeyi*. However, the intensity of the fusion protein bands was much fainter than that of the control Ls8-7 for which, according to our previous results [16], only a single copy of the *TeTr*CBHI gene was integrated into the genome. For example, the intensity of Western blot of the best strain (Ls11-7) only showed a density of about 30% of that of Ls8-7. Figure 2b shows the expression of the Fusion 2 construct where seven out of eight transformants presented positive Western blot bands, indicating that Fusion 2 could be successfully expressed in *L. starkeyi*. The signal density of the positive bands was different among these transformants with some of them being significantly higher than that of the control Ls8-7. For example, Western blot density of Ls12-18 is 4.6 times that of Ls8-7 demonstrating that the GH5 module of *Tr*EGII could enhance the secretion of *TeTr*CBHI. Ls12-18 of Fusion 2 was selected for subsequent analysis. Figure 2c shows the expression of Fusion 3 where five out of eight transformants showed positive bands in Western blot. Band density also varied among the transformants with Ls13-48 density being 3.2 times that of the control Ls8-7. This result suggests that the full-length *Tr*EGII can enhance the secretion of *TeTr*CBHI and verifies that the GH5 domain of TrEGII is the part that enables the improved secretion of the fusion protein constructs. Ls13-48 of Fusion 3 was selected for subsequent analysis. Only one copy of the Fusion 2 and 3 in transformants Ls12-18 and Ls13-48, respectively, was confirmed to be integrated in the genome by real-time quantitative PCR (qPCR) (Additional file 3).

### Endoglucanase activity of the *L. starkeyi* secretome by Congo red

Congo red staining of CMC was used to measure endoglucanase activity in the secretomes of the *L. starkeyi* transformants having integrated fusion protein genes. The individual proteins, *Tr*EGII and *TeTr*CBHI, were also assayed by Congo red staining (Fig. 3). As expected, *Tr*EGII, the positive control, exhibited high endoglucanase activity, whereas *TeTr*CBHI, the negative control, had very low activity. Clearly, only Fusion 3 (transformant LS13-48) showed strong endoglucanase activity similar to that of *Tr*EGII. This result shows that the full-length *Tr*EGII retained its activity in the Fusion 3 protein. The two fusion proteins, Fusion 1 and 2, showed very low activity. Surprisingly, the GH5 domain of *Tr*EGII did not display high activity in the Fusion 2 protein.

### *L. starkeyi* secretome activities of the fusion proteins on Avicel

The secretome activities measured on Avicel with no additional endoglucanase added are shown in Fig. 4. Among the three secretomes produced by the transformants, the activity of Fusion 3 is the highest, and Fusion 1 is the lowest. Fusion 2 showed a lower activity than Fusion 3, although more fusion protein was secreted (Fig. 2b). Activity of Fusion 3 was much higher than that of the individual *TeTr*CBHI, demonstrating that the activity of the secretome of *L. starkeyi* on Avicel can be enhanced by the fusion of *Tr*EGII with *TeTr*CBHI, relative to *TeTr*CBHI alone.

### Fusion 3 (*Tr*EGII–*TeTr*CBHI) is the most productive construct

The Congo red CMC staining assay clearly shows that endoglucanase activity is present only when the complete enzyme *Tr*EGII is linked to the N-terminal end of the *TeTr*CBHI. Surprisingly, no endoglucanase activity was observed when only the catalytic GH5 module of *Tr*EGII was linked with *TeTrCBHI,* as described above. While the CBM1 module of *Tr*EGII might be necessary for endoglucanase activity, the more likely explanation is that it is needed for proper folding of the fusion proteins. This theory is supported by the Avicel assays that show all other fusion constructs perform more poorly than *TeTr*CBHI and much worse than Fusion 3 (Fig. 4). For the Congo red CMC staining assay results—only the fusion construct with endoglucanase activity (Fig. 3) and the Avicel digestion assay (highest level of conversion, Fig. 4) suggest that Fusion 3 is the only reasonable choice to achieve both endoglucanase and cellobiohydrolase activities. While our main goal is to improve active CBHI expression, it is still beneficial to retain some endoglucanase activity and Fig. 4 clearly shows that Fusion 3 has much higher levels of Avicel conversion (more active CBHI) compared to the other fusion constructs. This is despite its lower level of expression compared to Fusion 2 (Fig. 2b). It should be noted here that protein expression levels measured using Western blot (such as that shown in Fig. 2) can include both active and inactive forms of CBHI [17].

**Fig. 5:**
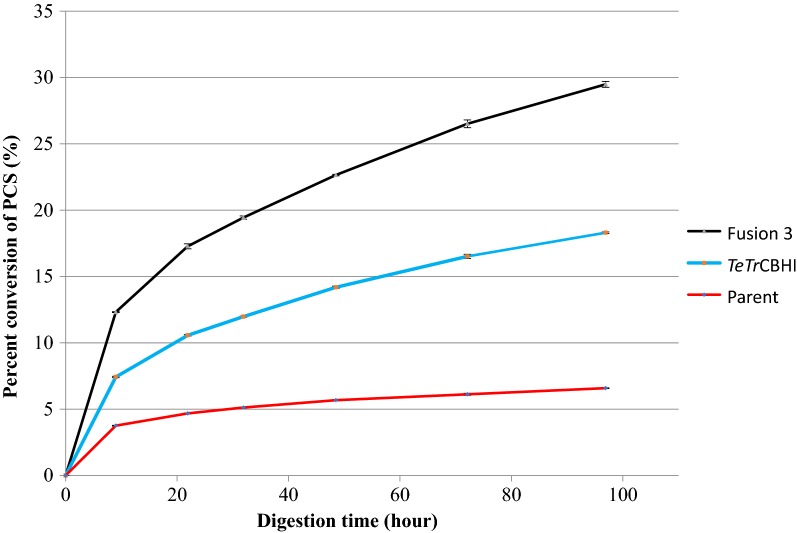
*L. starkeyi* secretome activity against PCS with added E1 endoglucanase. Each secretome protein was loaded at 84 mg of overall secretome protein, along with 2 mg endoglucanase E1 (*A. cellulolyticus* E1-CD Y245G mutant) and 0.5 mg purified *Aspergillus niger* β-glucosidase per g PCS glucan. The enzymes acted on 5 mg PCS glucan per mL, at 40 °C, pH 5.0 in 20 mM acetate. Conversion percentages were calculated based on HPLC determinations of released monomeric glucose

To further test the performance of this construct at the secretome level using a more industrially relevant substrate, we assayed the digestion of dilute acid pretreated corn stover (PCS) and compared it to the *L. starkeyi* secretome expressing just *TeTr*CBHI (Fig. 5). These PCS digestion assays reveal a significant improvement compared to the *TeTr*CBHI secretome. It should be noted that this assay was performed with added E1 endoglucanase that, due to the removal of cellobiose and prevention of inhibition by this glucose dimer, emphasizes the activity of the cellobiohydrolase. This result suggests that *L. starkeyi* can be expected to grow better on cellulosic biomass when expressing the Fusion 3 construct compared to the nonfused *TeTr*CBHI and an endoglucanase. However, these results do leave questions regarding the effect of different endoglucanases and does not clarify whether the improvement is due to increased activity or secretion level.

### Purified enzyme activity assays

**Fig. 6:**
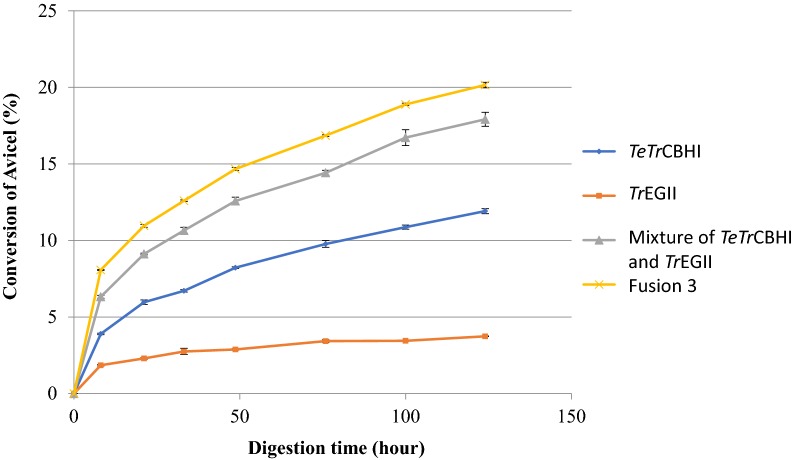
Digestion of Avicel with purified *L. starkeyi* enzymes. All enzymes used in this assay were expressed and purified from *L. starkeyi* and loaded at equimolar concentrations, *TeTr*CBHI at 16.72, *Tr*EGII at 13.28, and Fusion 3 at 31.24 mg/g cellulose. In addition, *A. niger* β-glucosidase was added at 1.0 mg/g cellulose. The assay enzymes were loaded on 5 mg Avicel/mL at 40 °C and pH 5.0 in 20 mM acetate buffer. Conversion percentages were calculated based on HPLC determinations of released monomeric glucose

To understand whether the increased conversion seen from the Fusion 3 secretome experiment is due to specific activity improvement in the fused proteins, we purified all the individual and fusion enzymes from *L. starkeyi*. First, we repeated the Avicel digestion assay with different combinations of the purified enzymes (Fig. 6). Fusion 3 showed significant improvement over a mixture of *Tr*EGII and *TeTr*CBHI, implying that intramolecular synergy does exist in Fusion 3.

**Fig. 7:**
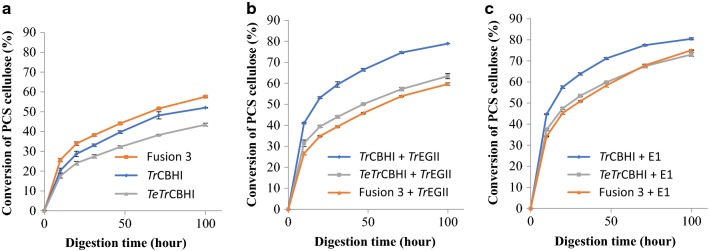
Digestion of PCS with purified *L. starkeyi* enzymes. **a** No added endoglucanase, **b** additional *Tr*EGII, and **c** additional E1. Each cellobiohydrolase was loaded at the molar equivalent of 25 mg of the reference *Tr*CBHI per g of biomass glucan. Endoglucanase (when present) was loaded at the molar equivalent of *A. cellulolyticus* E1-CD Y245G mutant at 2 mg/g substrate glucan. *A. niger* β-glucosidase was loaded in all assays at 0.5 mg/g substrate glucan. Conversion percentages were calculated based on HPLC determinations of released monomeric glucose. All enzymes used were purified

Fusion 3 achieves the highest PCS conversion when compared with *Tr*CBHI and *TeTr*CBHI without added endoglucanase (Fig. 7a). However, when either *Tr*EGII or E1 endoglucanase is added to the digestion mixture, *Tr*CBHI reaches a significantly higher PCS conversion level, while Fusion 3 and *TeTr*CBHI have a similar extent of conversion (Fig. 7b, c). The similar conversion level between Fusion 3 and *TeTr*CBHI with added endoglucanase is to be expected because the lack of endoglucanase is corrected for *TeTr*CBHI and further verifies that both *Tr*EGII and *TeTr*CBHI are functional in the fusion construct. These results agree with our previous results [16] reinforcing the conclusion that the fusion construct has not lost any cellobiohydrolase activity due to addition of *Tr*EGII to its N-terminal end.

**Fig. 8:**
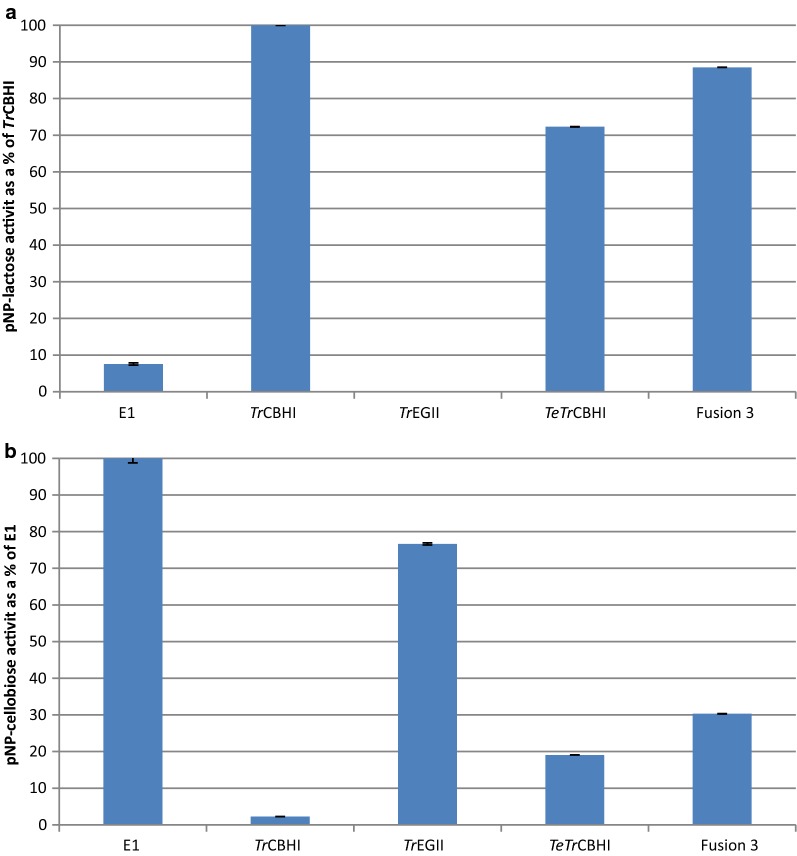
Activities of *L. starkeyi* purified enzymes on **a** *p*NP-lactose and **b** *p*NP-cellobiose. All enzymes were purified at equimolar loading (equal moles of fusion protein compared to each individual enzyme). E1 indicates *E. coli*-expressed E1 (*A. cellulolyticus* E1-CD Y245G mutant)

To better understand the effects of the fusion construct on the activity of the individual catalytic modules, we assayed these modules with the soluble substrates, *p*NP-lactose and *p*NP-cellobiose (Fig. 8). *p*NP-lactose is expected to be converted by the CBHI module, an exoglucanase, and not by the GH5 module of *Tr*EGII, an endoglucanase, whereas *p*NP-cellobiose is converted by GH5 and not by CBHI. Exoglucanase activity of the positive control native *Tr*CBHI against *p*NP-lactose showed a high activity, whereas the negative control, *A. cellulolyticus* E1, had low activity (Fig. 8a). Compared to the *L. starkeyi* expressed *TeTr*CBHI, the activity of Fusion 3 was increased by 22.4%, suggesting that the relative specific activity of the “embedded” CBHI domain in the Fusion 3 construct was enhanced significantly. This could indicate that a higher fraction of CBHI fusion proteins is in an active form in the fusion construct compared to the free enzyme. While higher fraction of active protein is not the same as improved catalytic rate, it still is an aspect of specific activity that is affected by the level of purification.

The endoglucanase activity of the positive control, E1 on *p*NP-cellobiose, exhibited high activity, whereas the negative control, native *Tr*CBHI, showed low activity (Fig. 8b). Compared to *L. starkeyi* expressed *Tr*EGII, the activity of Fusion 3 was reduced by 60.5% without deducting the compounding activity of *TeTr*CBHI. This result strongly suggests that the catalytic activity of the embedded GH5 module in Fusion 3 is significantly lower than its native form (Fig. 8b).

### Endoglucanase compatibility and cooperativity with different cellobiohydrolase constructs

**Fig. 9:**
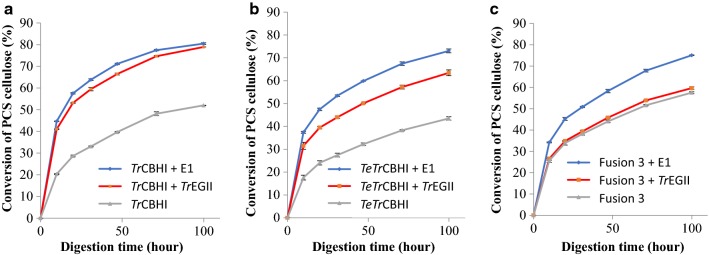
Digestion of PCS with purified *L. starkeyi* enzymes. Endoglucanase compatibility and cooperativity with different cellobiohydrolase constructs. **a** *Tr*CBHI, **b** *TeTr*CBHI, and **c** Fusion 3. Each cellobiohydrolase was loaded at the molar equivalent loading of 25 mg of the reference *Tr*CBHI per g of biomass glucan. Endoglucanase (when present) was loaded at the molar equivalent of a loading of *A. cellulolyticus* E1-CD Y245G mutant at 2 mg/g substrate glucan. *A. niger* β-glucosidase was loaded in all assays at 0.5 mg/g substrate glucan. Conversion percentages were calculated based on HPLC determinations of released monomeric glucose

To understand the differences between the two endoglucanases used in our experiments, *A. cellulolyticus* E1 and *Tr*EGII, we performed PCS digestion assays for all three cellobiohydrolases used in this study. *Tr*CBHI worked well with both endoglucanases, reaching higher conversion levels slightly earlier with E1 and both reaching 80% conversion after 100 h (Fig. 9a). However, for *TeTr*CBHI, adding E1 instead of *Tr*EGII results in a large improvement (Fig. 9b). As to be expected, E1 is also better with Fusion 3 and adding *Tr*EGII confers no benefits (Fig. 9c) again showing that the linked enzymes are fully functional.

### Fusion 3 construct in *S. cerevisiae* and *Y. lipolytica*

**Fig. 10:**
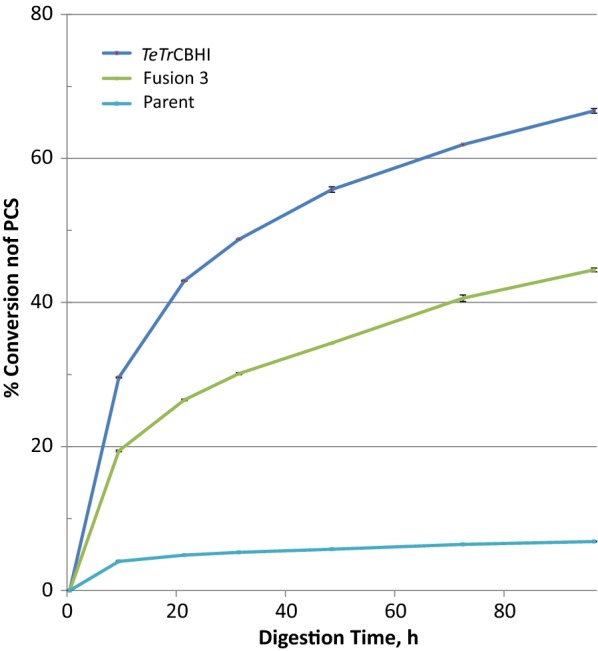
Saccharification of the cellulose content of PCS by the secretomes of *Y. lipolytica.* Secretome protein loaded at 84 mg/g biomass glucan, along with 2 mg/g *A. cellulolyticus* E1-CD Y245G mutant endoglucanase and 0.5 mg/g *A. niger* β-glucosidase acting on 5 mg/mL PCS. Digestions at 40 °C, pH 5.0 in 20 mM acetate

To understand how our results observed in *L. starkeyi* extend to yeasts in general, we expressed the Fusion 3 construct in *S. cerevisiae* and *Y. lipolytica.* Unfortunately, *p*NP-lactose assays of the *S. cerevisiae* secretome showed no observed activity (data not shown), whereas the *p*NP-cellobiose assay revealed only minimal activity for two of the expression constructs (Additional file 4). This result suggests that the Fusion 3 construct has trouble folding correctly and/or being secreted when expressed in *S. cerevisiae*, even though 11 different secretion signal peptides were tried. However, the Fusion 3 construct secreted from *Y. lipolytica* was functional but yielded lower secretome PCS conversion rates when compared with the unlinked *TeTr*CBHI in the presence of E1 (Fig. 10). This result raises a concern that in *Y. lipolytica*, Fusion 3 has a lower expression level or that much of the expressed enzyme is inactive.

To compare the secretion level of the Fusion 3 construct with that of *Tr*EGII alone in *Y. lipolytica*, a more thorough Western blot analysis was conducted, using both anti-CBHI and anti-EGII antibodies (Additional file 5). These results demonstrated that whereas Fusion 3 expressed in *Y. lipolytica* has a dramatically higher secretion level than that in a strain expressing *TeTr*CBHI alone (Additional file 5B, lanes 3 versus 2; approximately eightfold difference), the fusion protein secretion level is comparable to that of a strain expressing *Tr*EGII alone (Additional file 5C, lanes 3 versus 4). These data indicate that the *Tr*EGII component in the Fusion 3 construct is the determinant of secretion level of the fusion protein. Clearly, the lower expression level is not what caused the lower secretome level of PCS conversion shown in Fig. 10. This outcome leaves lower specific activity and/or damaged/inactive enzyme as possible explanations for the decreased PCS conversion.

### Fusion 3 performance in yeasts

**Fig. 11:**
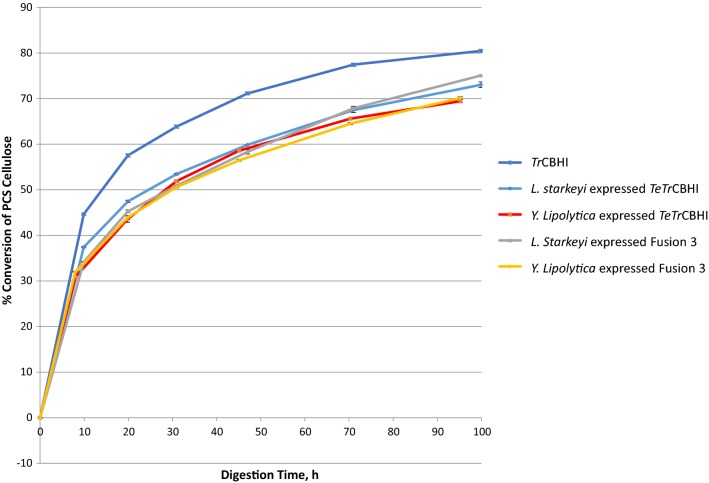
Comparison of PCS digestion with enzymes purified from *L. starkeyi* and *Y. lipolytica*. PCS was loaded at 5 mg glucan per mL pH 5.0 in 20 mM acetate, 40 °C. CBHI enzymes at molar equivalent of *T. reesei* CBHI loaded at 25 mg/g cellulose. Endoglucanase (*A. cellulolyticus* E1 cd Y245G mutant) at 2 mg/g cellulose and β-glucosidase (*A. niger*) at 0.5 mg/cellulose

So far, our experiments have shown that the Fusion 3 construct that combines both *Tr*EGII and the chimeric *TeTr*CBHI can significantly improve the yields of active cellobiohydrolase in *L. starkeyi*. Disappointingly, we were not able to express active fusion enzyme in *S. cerevisiae* and the PCS conversion extent of the *Y. lipolytica* secretome expressing the fusion protein was lower than for *TeTr*CBHI despite significantly increased expression levels (250 mg/L for Fusion 3 versus 32 mg/L for *TeTr*CBHI, estimated from Additional file 5 as in [15]). Expression trends in *Y. lipolytica* are good examples of yeast CBHI expression problems. High secretome level expression yield does not necessarily mean that all the expressed enzyme is active. This is in line with what has been reported previously [17, 27, 40]. To better understand the activity of Fusion 3 construct in *Y. lipolytica*, we performed a PCS digestion assay with purified *Y. lipolytica* and *L. starkeyi* Fusion 3 proteins and *Tr*CBHI in the presence of endoglucanase, E1 (Fig. 11). The results, as expected, show higher activity for *Tr*CBHI compared to the fusion protein expressed in both yeasts. However, the extent of conversion for the fusion constructs is reasonably high to expect these enzymes to work well in the context of yeast growing on lignocellulose biomass.

The differences between Fusion 3 proteins in *L. starkeyi* and *Y. lipolytica* are small (Fig. 11, extent of conversion: ~ 70% versus ~ 75%, respectively). This finding combined with the observation that *Y. lipolytica* expression level is high (Additional file 5) and the secretome activity against PCS (Fig. 10) is not better than with *TeTr*CBHI, leading us to conclude that the purified enzyme is fully functional. Likely, much of the initially expressed Fusion 3 enzyme in *Y. lipolytica* is misfolded or damaged leading to lower secretome activity. Peptide misfolding and incorrect disulfide bond formation are well-characterized problems for cellulase expression in yeasts [17, 41, 42]. However, when we have a closer look at the *L. starkeyi* and *Y. lipolytica* secretome assays with PCS (Figs. 5, 10), the level of conversion is higher for *Y. lipolytica* (50% versus 29%). This result suggests, together with our conclusions above, that the lower extent of conversion for Fusion 3 in *L. starkeyi* is due to lower expression levels.

### Enhancement of CBHI secretion in yeasts

CBHI enzymes have been recognized as the most functional enzymes in the fungal cellulase systems in nature [43, 44] and are therefore the primary consideration to be expressed in yeasts to create new CBP microorganisms. *cbh1* genes could be expressed in some yeasts successfully, but problems, such as low yield of their recombinant proteins and low activities of those enzymes caused by low secretion and hyper-glycosylation, have affected their activities [17, 27, 45]. To overcome these barriers, strong signal peptides, higher-functional promoters, and modified endoplasmic reticulum have been used to achieve enhanced CBHI expression in yeasts [17, 46]. Although these approaches have achieved some progress, results have not been completely satisfactory.

The Avicel assay in Fig. 4 was conducted with equal protein loading of all three fusion constructs (28 mg of overall secretome protein) to compare secretome-specific activity versus total protein. The results of this experiment show that Fusion 3 has a much higher level of Avicel deconstruction (secretome-specific activity) than Fusions 1 and 2 even though Fusion 2, with a leading GH5 module, has a higher overall expression level (Fig. 2). This result suggest strongly that the Fusion 3 construct produces more active protein compared to the other fusion constructs. Furthermore, the protein abundance of Fusion 3 at the secretome level indicated by Western blot was 3.2-fold higher compared to the chimeric *TeT*rCBHI (Fig. 2c). To put this in perspective, we routinely observe a threefold to tenfold yield increase of purified active Fusion 3 compared to *TeTr*CBHI from *L. starkeyi*. Thus, Fusion 3 (with the leading *Tr*EGII enzyme) results in better expression of active CBHI from *L. starkeyi* compared to the other fusion constructs tested, as well as chimeric *TeTr*CBHI.

Bacterial multi-functional cellulases widely exist in nature, but few are found in fungal cellulase systems [25, 47]. Some artificial fungal multi-functional cellulases were designed and characterized recently [48–50]. In this study, we used an easily secreted protein *Tr*EGII to lead the more difficult to secrete chimeric *TeTr*CBHI. We found that Fusion 3 construct (*Tr*EGII–*TeTr*CBHI) had an increased secretion level of at least threefold in *L. starkeyi* compared to that of individual chimeric *TeTr*CBHI. The same benefits did not extend to *Y. lipolytica* or *S. cerevisiae,* indicating that this approach is not universally applicable. The likely reason for this outcome is continued expression and/or folding problems in these yeasts that the fusion construct was designed to prevent. Although expression improvements in the Fusion 3 construct do not seem to be universal, we identified other benefits. With soluble substrates, the exoglucanase activity of the embedded GH7A domain of Fusion 3 was significantly higher than that of the individual GH7A in *TeTr*CBHI (Fig. 8). It is also noteworthy that we showed the GH5 module of *Tr*EGII to be the required protein domain for increased secretion in *L. starkeyi* (Fig. 4). The approach of enhancing CBHI secretion or activity by adding well expressing protein domains may, in specific cases, be useful for production of other proteins in yeasts.

## Conclusions

In conclusion, we have used an easily secreted protein, *Tr*EGII, to lead the more difficult to secrete chimeric *TeTr*CBHI to explore possible expression level improvements of this fusion protein strategy in the industrially relevant yeasts: *S. cerevisiae, Y. lipolytica,* and *L. starkeyi*. We showed the GH5 module of *Tr*EGII to be the required protein domain for increased secretion in *L. starkey*i. We also showed that the Fusion 3 construct (*Tr*EGII–*TeTr*CBHI) had an increased secretion level of at least threefold compared to that of individual chimeric *TeTr*CBHI and that the purified fusion protein had significantly higher specific activity against *p*NP-lactose. Clearly, the fusion 3 construct greatly improves active CBHI secretion in *L. starkeyi*. However, the same benefits did not extend to *Y. lipolytica* or *S. cerevisiae,* indicating that this approach is not universally applicable. Expression of the Fusion 3 construct in *S. cerevisiae* was poor, and only minimal activity was observed on the *p*NP-cellobiose substrate, whereas no activity was observed for *p*NP-lactose hydrolysis, indicating that *Tr*EGII may have folded correctly; however, the CBHI module was inactive.

In *L. starkeyi*, the exoglucanase activity (measured with soluble substrates) of the embedded GH7A module of Fusion 3 was significantly higher than that of the individual GH7A in *TeTr*CBHI. The fact that active CBHI secretion and consequently specific activity was improved shows that this fusion construct engineering strategy can work in yeasts and possibly other organisms. Increased secretion levels and specific activity are beneficial not only for CBP-biofuels pursuits, but more broadly for the general secretion of enzymes from yeast.

## Additional files

**Additional file 1. ** Primers used in this study.

**Additional file 2.** Plasmids used in this study. *Tr*EGII indicates the *T. reesei* EGII, *TeTr*CBHI for a chimeric CBHI generated by fusion of the catalytic module from *Talaromyces emersonii* CBHI with the linker peptide and cellulose-binding module from *T. reesei* CBHI. Lspyk represents native *L. starkeyi* pyruvate kinase promoter, Lsgal1 for the *L. starkeyi* galactokinase terminator, spDEXII represent native signal peptide of *L. starkeyi* dextranase 2. ScTEF = *S. cerevisiae* translation elongation factor 1 promoter. ScCYC1 = *S. cerevisiae* cytochrome b-c1 complex terminator.

**Additional file 3. ** Further information regarding the *L. starkeyi* genetic transformation system and screening of colonies.

**Additional file 4.** *S. cerevisiae* secretome Fusion 3 construct screening with pNP-cellobiose. A) activity among *S. cerevisiae* constructs having different secretion signal peptides. B) Activity compared to the Fusion 3 construct expressed in *L. starkeyi*. pControl is a negative control empty vector.

**Additional file 5.** Comparison of protein secretion levels between Fusion 3 expressing *Y. lipolytica* transformants and the individual *Tr*EGII or *TeTr*CBHI expressing transformants by using SDS-PAGE, Western blot and densitometric analyses. A) SDS-PAGE gel. B) Western blot with anti-*Tr*CBHI antibody. C) Western blot with anti-*Tr*EGII antibody. While chimeric *TeTr*CBHI and its Western blot bands are indicated by red text and arrows, respectively, *Tr*EGII and its Western blot bands are indicated by green text and arrows, respectively

## Abbreviations

CBP: consolidated bioprocessing
CBHI: cellobiohydrolase I
*Tr*EGII: *T. reesei* EGII
*TeTr*CBHI: a chimeric CBHI generated by the fusion of the catalytic module from *Talaromyces emersonii* CBHI with the linker peptide and cellulose-binding module from *T. reesei* CBHI
Fusion 3, *Tr*EGII–*TeTr*CBHI: *Tr*EGII linked to *Tr*CBHI
VVM: volume of gas per volume of media per minute
PCS: dilute acid-pretreated corn stover
