# Supplementary material for: Expression of an endoglucanase–cellobiohydrolase fusion protein in Saccharomyces cerevisiae, Yarrowia lipolytica, and Lipomyces starkeyi
Source: Biotechnol Biofuels. 2018 Dec 3;11:322. doi: 10.1186/s13068-018-1301-y (PMC6278004; doi:10.1186/s13068-018-1301-y)
Supplement: Supplementary file 2 — Additional file 2. Plasmids used in this study. TrEGII indicates the T. reesei EGII, TeTrCBHI for a chimeric CBHI generated by fusion of the catalytic module from Talaromyces emersonii CBHI with the linker peptide and cellulose-binding module from T. reesei CBHI. Lspyk represents native L. starkeyi pyruvate kinase promoter, Lsgal1 for the L. starkeyi galactokinase terminator, spDEXII represent native signal peptide of L. starkeyi dextranase 2. ScTEF = S. cerevisiae translation elongation factor 1 promoter. ScCYC1 = S. cerevisiae cytochrome b-c1 complex terminator. [file 13068_2018_1301_MOESM2_ESM.pptx]

## Slide 1
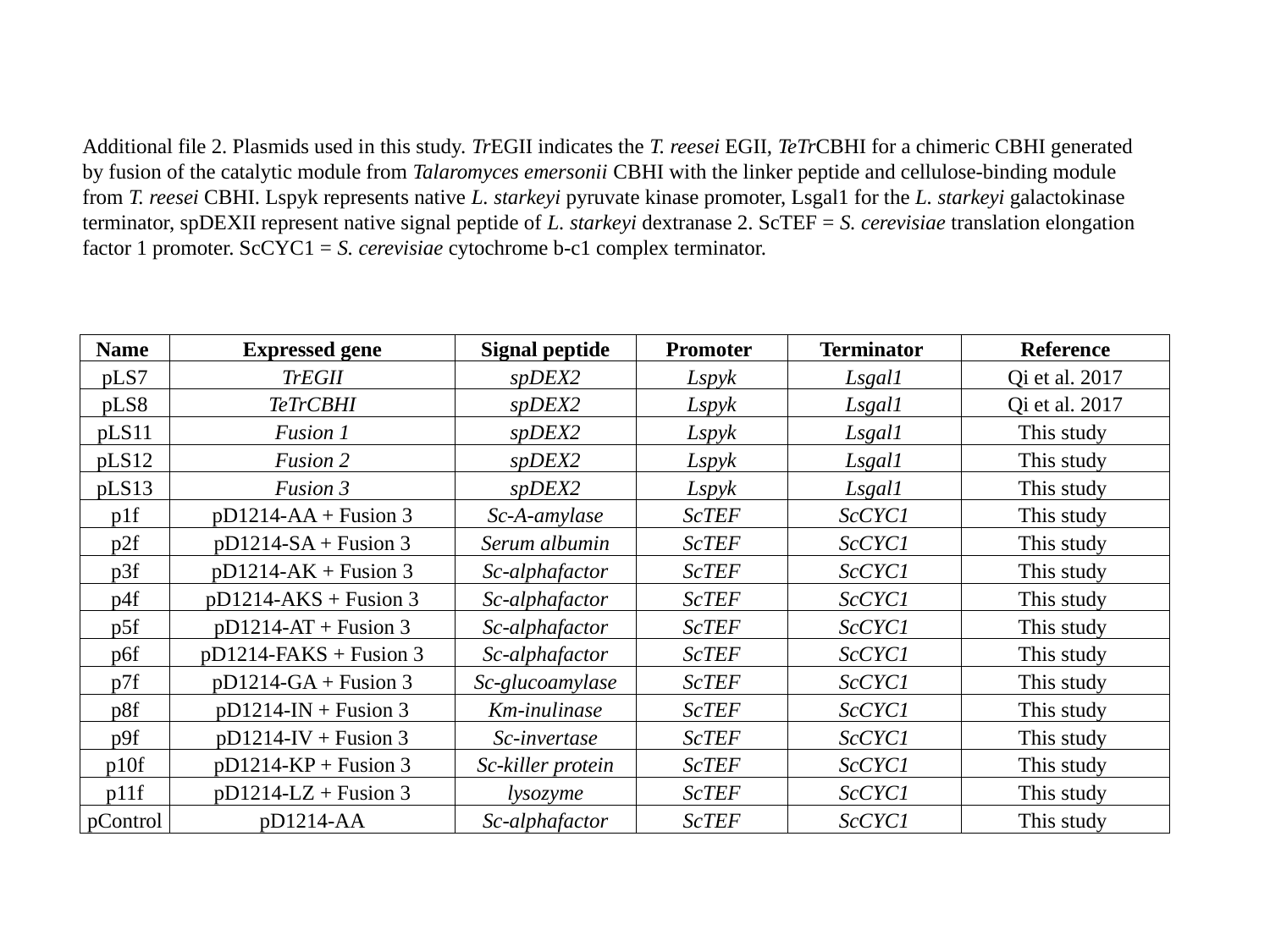

Additional file 2. Plasmids used in this study. TrEGII indicates the T. reesei EGII, TeTrCBHI for a chimeric CBHI generated by fusion of the catalytic module from Talaromyces emersonii CBHI with the linker peptide and cellulose-binding module from T. reesei CBHI. Lspyk represents native L. starkeyi pyruvate kinase promoter, Lsgal1 for the L. starkeyi galactokinase terminator, spDEXII represent native signal peptide of L. starkeyi dextranase 2. ScTEF = S. cerevisiae translation elongation factor 1 promoter. ScCYC1 = S. cerevisiae cytochrome b-c1 complex terminator.
| Name | Expressed gene | Signal peptide | Promoter | Terminator | Reference |
| --- | --- | --- | --- | --- | --- |
| pLS7 | TrEGII | spDEX2 | Lspyk | Lsgal1 | Qi et al. 2017 |
| pLS8 | TeTrCBHI | spDEX2 | Lspyk | Lsgal1 | Qi et al. 2017 |
| pLS11 | Fusion 1 | spDEX2 | Lspyk | Lsgal1 | This study |
| pLS12 | Fusion 2 | spDEX2 | Lspyk | Lsgal1 | This study |
| pLS13 | Fusion 3 | spDEX2 | Lspyk | Lsgal1 | This study |
| p1f | pD1214-AA + Fusion 3 | Sc-A-amylase | ScTEF | ScCYC1 | This study |
| p2f | pD1214-SA + Fusion 3 | Serum albumin | ScTEF | ScCYC1 | This study |
| p3f | pD1214-AK + Fusion 3 | Sc-alphafactor | ScTEF | ScCYC1 | This study |
| p4f | pD1214-AKS + Fusion 3 | Sc-alphafactor | ScTEF | ScCYC1 | This study |
| p5f | pD1214-AT + Fusion 3 | Sc-alphafactor | ScTEF | ScCYC1 | This study |
| p6f | pD1214-FAKS + Fusion 3 | Sc-alphafactor | ScTEF | ScCYC1 | This study |
| p7f | pD1214-GA + Fusion 3 | Sc-glucoamylase | ScTEF | ScCYC1 | This study |
| p8f | pD1214-IN + Fusion 3 | Km-inulinase | ScTEF | ScCYC1 | This study |
| p9f | pD1214-IV + Fusion 3 | Sc-invertase | ScTEF | ScCYC1 | This study |
| p10f | pD1214-KP + Fusion 3 | Sc-killer protein | ScTEF | ScCYC1 | This study |
| p11f | pD1214-LZ + Fusion 3 | lysozyme | ScTEF | ScCYC1 | This study |
| pControl | pD1214-AA | Sc-alphafactor | ScTEF | ScCYC1 | This study |
