# Supplementary material for: Expression of an endoglucanase–cellobiohydrolase fusion protein in Saccharomyces cerevisiae, Yarrowia lipolytica, and Lipomyces starkeyi
Source: Biotechnol Biofuels. 2018 Dec 3;11:322. doi: 10.1186/s13068-018-1301-y (PMC6278004; doi:10.1186/s13068-018-1301-y)
Supplement: Supplementary file 3 — Additional file 3. Further information regarding the L. starkeyi genetic transformation system and screening of colonies. [file 13068_2018_1301_MOESM3_ESM.pdf]

The genetic transformation system of *L. starkeyi* has been established only recently, and so far this system can only be used to integrate genes into the host cell's genome randomly. It cannot perform targeted integration. Due to this technical limitation, the fusion genes descripted in this study were integrated into the genome randomly.

However, using the random insertion has the following issues, but not limited to: 1) fusion gene cassette may be damaged due to digestion from the host cells defense mechanisms, but its resistance gene cassette (clonNat in this study) remains intact; 2) the expression levels of the target gene may vary due to the positional effects as the gene is inserted at different locations in the genome, and 3) expression of target gene may be affected by multiple gene copy numbers. For these reasons, we investigated 18, 56 and 48 clonNAT resistant colonies by Western blots for each of the constructs, Fusions 1, 2 and 3, respectively although only 8 of them for each construct were shown in Figure 2. Indeed, as presented by newly added Table 1 (please see below), some colonies were resistant to clonNAT, but their Western blots were negative; whereas other colonies are both resistant to clonNAT and positive on Western blots. We identified a total of 9, 38 and 24 Western blot positive colonies for each of the constructs. Among the Western blot positive colonies, 0 (0 %), 22 (57.8%) and 14 (58.3 %) of the colonies showed greater abundance than the non-fusion *TeTr*CBHI control and therefore we think the results presented here are sufficient to support our conclusions.

We made the conclusion that "the observed expression differences are due to the arrangement of the protein domains" based on the following three observations (take Fusion 3 as an example):

- The size of the recombinant protein of Fusion 3 was as expected, indicating gene expression was correct.
- Only one copy of Fusion 3 gene exists in the genome (strain LS13-48) (see below).
- Greater than 50% of Fusion-3 transformants (58.3 %) showed higher abundance of their recombinant fusion proteins than that of the non-fusion *TeTr*CBHI control, detected by Western blot (Table 1).

The gene copy number of the Fusion 2 of transformant Ls12-18 was further analyzed by real-time quantitative PCR (qPCR). These results showed that for transformant Ls12-18, the copy-number (X_0/R_0) of the target gene (X) versus the reference gene (R, which is the single-copy house-keeping gene *eif5*) is nearly 1, indicating that transformant Ls12-18 harbored a single copy of the Fusion 2 gene, thus excluding the possibility that the secretion enhancement of Fusion 2 is due to multiple-copy insertion into the genome. The gene copy number of Fusion 3 in the genome of the transformant Ls13-48 was also analyzed by real-time qPCR, also showing that the copy number (X_0/R_0) for Fusion 3 gene in Ls13-48 is one.

Table 1. Colonies screened by both antibiotic and Western blot

		Number of Western Blot Positive Colonies		
Transformation	cNAT Resistant Colonies	Greater abundance than control	Equal or lesser abundance than control	Total
Fusion 1	18	0 (0)	9	9
Fusion 2	56	22 (57.8%)	16	38
Fusion 3	48	14 (58.3 %)	10	24

(Note: Control is individual *TeTr*CBHI)

The gene transcription levels vary among different chromosomal regions (Ref 1) and the location of integration of heterologous genes in genome; therefore, may influence the expression level of the heterologous gene. Indeed, differences of up to 8.7-fold for the lacZ reporter gene and 14-fold for the β-galactosidase gene expression between the lowest and highest expression have been found based on the location of integration in yeast genomes (Ref 2 and 3). In *L. starkeyi,* this potential location effect on heterologous gene expression has been reported as well (Ref 4). In this study, fusion genes were integrated into *L. starkeyi* genome randomly, and their integration positions in the genome likely affect their expressions, providing a major reason why some low expressing colonies were observed in Fusion 2 and Fusion 3. As described above and shown in Table 1, we screened 18, 56 and 48 colonies for each of the constructs and found that this effect seems well correlated with the nature of the multi-protein construct, i.e., fusion 2/fusion 3 versus fusion 1. Figure 2 only shows representative ones, not all constructs.

References:

1. Yamane S, Yamaoka M, Yamamoto M, Maruki T, Matsuzaki H, Hatano T, Fukui S: **Region specificity of chromosome III on gene expression in the yeast Saccharomyces cerevisiae**. J Gen Appl Microbiol. 1998, **44**:275–281.

2. Bai Flagfeldt D, Siewers V, Huang L, Nielsen J: **Characterization of chromosomal integration sites for heterologous gene expression in Saccharomyces cerevisiae**. Yeast. 2009, **26**:545–551.

3. Thompson A, Gasson MJ: **Location effects of a reporter gene on expression levels and on native protein synthesis in Lactococcus lactis and Saccharomyces cerevisiae**. Appl Environ Microbiol. 2001, **67**:3434–3439.

4. Xu Q, Knoshaug EP, Wang W, Alahuhta M, Baker JO, Yang S, Vander Wall T, Decker SR, Himmel ME, Zhang M, Wei H: **Expression and secretion of fungal endoglucanase II and chimeric cellobiohydrolase I in the oleaginous yeast Lipomyces starkeyi**. Microb Cell Fact. 2017, **16(1)**:126.
