# Supplementary material for: Expression of an endoglucanase–cellobiohydrolase fusion protein in Saccharomyces cerevisiae, Yarrowia lipolytica, and Lipomyces starkeyi
Source: Biotechnol Biofuels. 2018 Dec 3;11:322. doi: 10.1186/s13068-018-1301-y (PMC6278004; doi:10.1186/s13068-018-1301-y)

Additional file 4. *S. cerevisiae* secretome Fusion 3 construct screening with pNP-cellobiose. A) activity among *S. cerevisiae* constructs having different secretion signal peptides. B) Activity compared to the Fusion 3 construct expressed in *L. starkeyi*. pControl is a negative control empty vector.

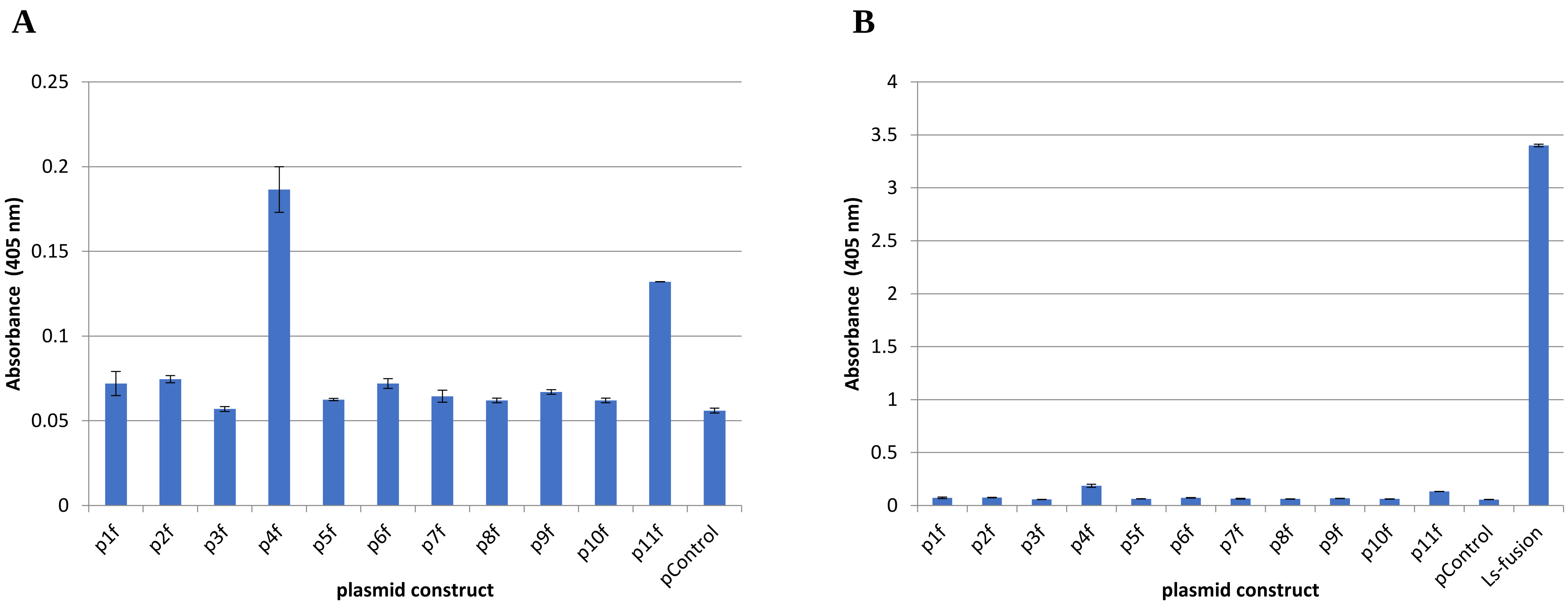

Supplement: Supplementary file 4 — Additional file 4. S. cerevisiae secretome Fusion 3 construct screening with pNP-cellobiose. A) activity among S. cerevisiae constructs having different secretion signal peptides. B) Activity compared to the Fusion 3 construct expressed in L. starkeyi. pControl is a negative control empty vector. [file 13068_2018_1301_MOESM4_ESM.pdf]
