# Supplementary material for: Expression of an endoglucanase–cellobiohydrolase fusion protein in Saccharomyces cerevisiae, Yarrowia lipolytica, and Lipomyces starkeyi
Source: Biotechnol Biofuels. 2018 Dec 3;11:322. doi: 10.1186/s13068-018-1301-y (PMC6278004; doi:10.1186/s13068-018-1301-y)
Supplement: Supplementary file 5 — Additional file 5. Comparison of protein secretion levels between Fusion 3 expressing Y. lipolytica transformants and the individual TrEGII or TeTrCBHI expressing transformants by using SDS-PAGE, Western blot and densitometric analyses. A) SDS-PAGE gel. B) Western blot with anti-TrCBHI antibody. C) Western blot with anti-TrEGII antibody. While chimeric TeTrCBHI and its Western blot bands are indicated by red text and arrows, respectively, TrEGII and its Western blot bands are indicated by green text and arrows, respectively [file 13068_2018_1301_MOESM5_ESM.pptx]

## Slide 1
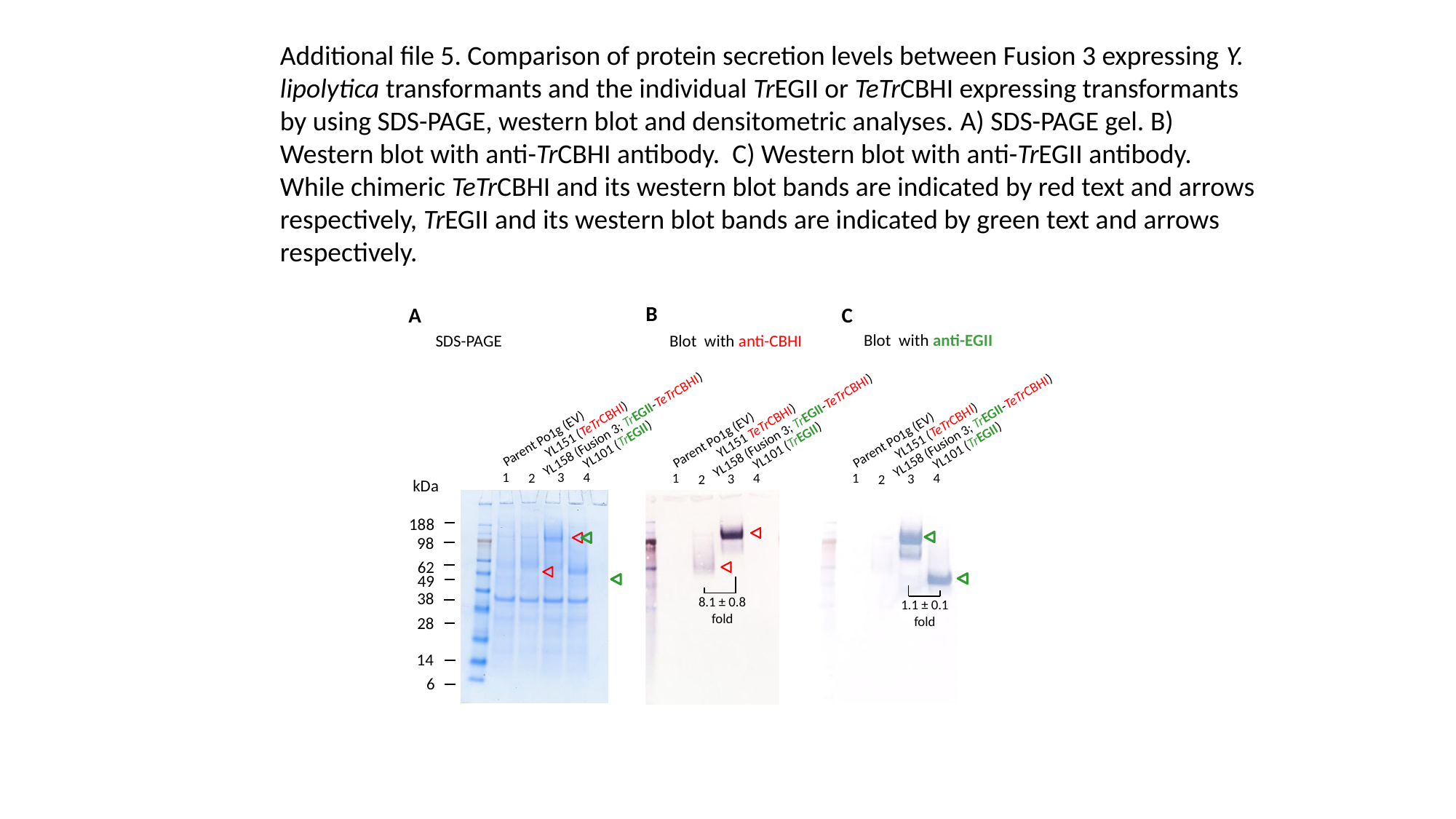

Additional file 5. Comparison of protein secretion levels between Fusion 3 expressing Y. lipolytica transformants and the individual TrEGII or TeTrCBHI expressing transformants by using SDS-PAGE, western blot and densitometric analyses. A) SDS-PAGE gel. B) Western blot with anti-TrCBHI antibody. C) Western blot with anti-TrEGII antibody. While chimeric TeTrCBHI and its western blot bands are indicated by red text and arrows respectively, TrEGII and its western blot bands are indicated by green text and arrows respectively.
B
C
A
Blot with anti-EGII
SDS-PAGE
Blot with anti-CBHI
YL158 (Fusion 3; TrEGII-TeTrCBHI)
YL151 (TeTrCBHI)
Parent Po1g (EV)
YL101 (TrEGII)
1
4
3
2
kDa
188
98
62
49
38
28
14
6
YL158 (Fusion 3; TrEGII-TeTrCBHI)
YL151 TeTrCBHI)
Parent Po1g (EV)
YL101 (TrEGII)
1
4
3
2
YL158 (Fusion 3; TrEGII-TeTrCBHI)
YL151 (TeTrCBHI)
Parent Po1g (EV)
YL101 (TrEGII)
1
4
3
2
8.1 ± 0.8
fold
1.1 ± 0.1
fold
